# Improved TEA Sensitivity and Selectivity of In_2_O_3_ Porous Nanospheres by Modification with Ag Nanoparticles

**DOI:** 10.3390/nano12091532

**Published:** 2022-05-02

**Authors:** Dengke Li, Yanwei Li, Xiaohua Wang, Guang Sun, Jianliang Cao, Yan Wang

**Affiliations:** 1 School of Materials Science and Engineering, Henan Polytechnic University, Jiaozuo 454000, China; denkolee@yeah.net; 2 The Collaboration Innovation Center of Coal Safety Production of Henan Province, Henan Polytechnic University, Jiaozuo 454000, China; caojianliang@hpu.edu.cn (J.C.); yanwang@hpu.edu.cn (Y.W.); 3 School of Chemistry and Chemical Engineering, Henan Polytechnic University, Jiaozuo 454000, China; daisy7803@126.com

**Keywords:** In_2_O_3_, porous nanospheres, Ag modification, TEA, gas sensor

## Abstract

A highly sensitive and selective detection of volatile organic compounds (VOCs) by using gas sensors based on metal oxide semiconductor (MOS) has attracted increasing interest, but still remains a challenge in gas sensitivity and selectivity. In order to improve the sensitivity and selectivity of In_2_O_3_ to triethylamine (TEA), herein, a silver (Ag)-modification strategy is proposed. Ag nanoparticles with a size around 25–30 nm were modified on pre-synthesized In_2_O_3_ PNSs via a simple room-temperature chemical reduction method by using NaBH_4_ as a reductant. The results of gas sensing tests indicate that after functionalization with Ag, the gas sensing performance of In_2_O_3_ PNSs for VOCs, especially for TEA, was remarkably improved. At a lower optimal working temperature (OWT) of 300 °C (bare In_2_O_3_ sensor: 320 °C), the best Ag/In_2_O_3_-2 sensor (Ag/In_2_O_3_ PNSs with an optimized Ag content of 2.90 wt%) shows a sensitivity of 116.86/ppm to 1–50 ppm TEA, about 170 times higher than that of bare In_2_O_3_ sensor (0.69/ppm). Significantly, the Ag/In_2_O_3_-2 sensor can provide a response (R_a_/R_g_) as high as 5697 to 50 ppm TEA, which is superior to most previous TEA sensors. Besides lower OWT and higher sensitivity, the Ag/In_2_O_3_-2 sensor also shows a remarkably improved selectivity to TEA, whose selectivity coefficient (S_TEA_/S_ethanol_) is as high as 5.30, about 3.3 times higher than that of bare In_2_O_3_ (1.59). The sensitization mechanism of Ag on In_2_O_3_ is discussed in detail.

## 1. Introduction

In recent decades, with the sustained growth of environmental issues, fast and timely detection of flammable and harmful gases in our surroundings has attracted increasing attention [1,2,3,4]. Triethylamine (TEA), as a member of volatile organic compounds (VOCs), is a typical organic amine compound with strong pungent smell, which has been widely used in industry as a catalyst, solvent, synthetic fuel, preservative, and so on [5,6]. However, owing to its toxic nature, long-term exposure to excessive TEA (>1 ppm) may cause a series of health problems, such as skin irritation, headache, gastroenteritis, and pulmonary edema [7,8]. In practice, some traditional techniques, such as chromatographic and colorimetric methods, have been applied to detect TEA, but usually suffer from high price, complicated operations, and time-consuming detection processes [9]. Therefore, developing fast and convenient techniques for TEA detection is of great important.

Among different state-of-the-art gas detection techniques, gas sensors based on metal oxide semiconductor (MOS) have attracted extensive attention because of their outstanding merits of low price, small volume, easy fabrication and integration, high sensitivity for diverse gases, and fast response speed [10,11,12,13]. Up to now, several MOSs such as SnO_2_ [14], ZnO [15], TiO_2_ [16], WO_3_ [17], In_2_O_3_ [18,19], and Fe_2_O_3_ [20] have been studied for TEA sensor application, and significant advances have been achieved. However, there are still some obstacles that need to be overcome from the perspective of practical application, especially in the aspects of sensitivity and selectivity. In previous studies, yearning to improve the gas sensing properties of MOS, researchers have attempted several sensitization strategies. These strategies mainly include fabrication of novel nanostructures [21], doping with foreign element [22,23], modification with noble metal (nanoparticles or single atoms) [24,25,26], and combination with other MOSs to construct heterojunction [27,28]. Among these strategies, modification with nano-sized noble metals was found to be very effective to upgrade the gas sensing performance of MOS, especially in the aspect of gas sensitivity. For example, Sukee et al. [29] prepared Ag-loaded LaFeO_3_ by a flame spray pyrolysis (FSP) method and found that the 0.1 wt% Ag-loaded LaFeO_3_ sensor showed a response of 60 to 100 ppm acetylene, almost 12 times higher than that of the pure LaFeO_3_ sensor. Cai et al. [30] fabricated a highly selective H_2_ sensor fabricated with Pd nanoparticle-decorated SnO_2_ nanowires, whose response to 100 ppm H_2_ was about 12.7 times higher than that of the bare SnO_2_ sensor. Yang et al. [31] reported that, benefitting from the increased concentration of oxygen vacancy after modification with Au, the Au-decorated In_2_O_3_ hollow nanosphere sensor showed excellent sensitivity (26.3 of 100 ppm) and selectivity toward 1-butylamine at the optimized working temperature of 340 °C. All of the above studies have demonstrated that during the gas sensing process, the noble metal sensitizer can bring a series of positive effects on MOS, such as facilitating the formation of chemisorbed oxygen [32,33], modulating the space charge layer [34,35,36], and catalyzing the gas sensing reaction between gaseous analyte and chemisorbed oxygen [37,38,39].

In_2_O_3_, as an n-type semiconductor (*E*_g_ = 3.55–3.75 eV) with diverse functions, has been widely studied in the fields of lithium ion battery [40], supercapacitor [41], photocatalyst [42], and gas sensor [43] due to its unique physical and chemical properties, such as low toxicity, strong inoxidizability, and high electric mobility [44]. For gas sensor applications, In_2_O_3_ has been found to be sensitive to various gases, including TEA [32], CO [45], methane [46], acetone [47], hydrogen [48], alcohol [49], NO2 [50], etc. To improve the gas sensitivity of In_2_O_3_ to TEA, different noble metals were used as sensitizers to functionalize In_2_O_3_. For example, Zheng et al. prepared porous In_2_O_3_ microspheres by annealing the In_2_S_3_ precursor and then decorated Au nanoparticles on them to obtain novel Au/In_2_O_3_ hybrid microspheres. At an operating temperature of 280 °C, the hybrid Au/In_2_O_3_ microspheres showed a response of 648.2 to 100 ppm TEA, higher than that of pristine In_2_O_3_ [51]. Liu et al. reported a TEA sensor that fabricated with Pd nanoparticles-functionalized In_2_O_3_ microspheres, whose response to 50 ppm TEA at 220 °C was 47.56, higher than that of In_2_O_3_ counterpart [52]. Compared with other noble metals (Au, Pt, Pd), Ag is more suitable for large-scale practical application owing to its lower price. Although Ag has been proven to be a valid sensitizer for In_2_O_3_ to sense NO_2_ [53] and H_2_S [54], there are few reports, to the best of our knowledge, on improving the TEA sensing performance of In_2_O_3_ by modification with Ag, as well as the detailed sensitization effects of Ag on In_2_O_3_ for sensing TEA.

In this study, Ag nanoparticles with a size of around 25–30 nm were evenly decorated on pre-synthesized In_2_O_3_ porous nanospheres (PNSs) via a simple room-temperature chemical reduction method. To check the influence of Ag modification on In_2_O_3_ and further understand the sensitization effect of Ag nanoparticles, the prepared bare and Ag-modified In_2_O_3_ samples were characterized by various techniques and their TEA sensing performances were investigated in detail. Research results indicate that after modification with Ag, the In_2_O_3_ sensor showed impressive improvements in TEA sensing performance, especially of lower operating temperature, much higher sensitivity, and better selectivity. Specifically, at its optimal working temperature (300 °C), the best Ag/In_2_O_3_ sensor can give a response as high as 5697 to 50 ppm TEA, which is about 158 times higher than that of bare In_2_O_3_ sensor (36) and is also superior to most previous TEA sensors. The upgraded gas sensing performances of Ag/In_2_O_3_ sensor can be ascribed to the spillover and catalytic effects of Ag nanoparticles, as well as the electronic sensitization effect of the Ag–In_2_O_3_ Schottky junction. Our research not only provides a promising sensitization strategy to enhance the TEA sensing performance of In_2_O_3_, but also contributes to a deeper understanding on the sensitization mechanism of Ag/In_2_O_3_ sensor.

## 2. Materials and Methods

### 2.1. Sample Preparation

All chemicals applied in our experiment were of analytical grade and used as received without further purification. To synthesize Ag-modified In_2_O_3_ PNSs, bare In_2_O_3_ PNSs were synthesized in advance according to our previous method [55]. Briefly, 0.42 g of In(NO_3_)_3_·4.5H_2_O and 0.71 g of sodium citrate were dissolved in 80 mL distilled water under magnetic stirring. After adding 0.20 g of urea into above solution, the obtained reaction solution was sealed in a Teflon-lined autoclave with the capacity of 100 mL and heated at 160 °C for 24 h. After reaction, the product was collected by centrifugation, washed with distilled water and absolute ethanol alternately, and dried at 60 °C in air for 8 h. Finally, bare In_2_O_3_ sample was obtained by annealing the collected powder at 500 °C (heating rate: 2 °C/min) in air for 3 h.

To synthesize Ag-modified In_2_O_3_ PNSs, a designed amount of as-prepared bare In_2_O_3_ powder was ultrasonically dispersed into 150 mL of AgNO_3_ aqueous solution (0.6 mmol/L), followed by adding 13 mL of NaBH_4_ (7 mmol/L) aqueous solution drop by drop. After the mixed solution was sonicated at room temperature for 3 h, dark brown precipitates were collected and purified by washing with distilled water. Finally, the precipitate was dried at 60 °C for 8 h to obtain the final Ag-modified In_2_O_3_ sample. The Ag content in Ag-modified In_2_O_3_ sample was controlled by adjusting the using amount of bare In_2_O_3_. When the mass of In_2_O_3_ was set as 88.26, 74.68, and 64.72 mg, Ag/In_2_O_3_ samples with theoretical Ag contents of 11 wt%, 13 wt%, and 15 wt% were prepared and denoted as Ag/In_2_O_3_-1, Ag/In_2_O_3_-2, and Ag/In_2_O_3_-3, respectively.

### 2.2. Characterizations

The phase structure analysis of the samples was performed by powder X-ray diffraction (XRD, Bruker D8, Cu-Kα1 radiation, λ = 1.5418 Å) in the 2θ range of 10–90°. The actual Ag contents in Ag/In_2_O_3_ samples were measured by using an inductively coupled plasma optical emission spectrometer (ICP-OES, Agilent ICP-OES 725 ES). The morphology and microstructure of the samples were characterized by scanning electron microscope (SEM, Merlin Compact) and transmission electron microscope (TEM, JEOL JEM 2100 F). The chemical composition and surface state of the materials were analyzed by X-ray photoelectron spectroscopy (XPS, Thermo SCIENTIFIC ESCALAB 250Xi). The binding energy of elements is calibrated with the surface adventitious carbon (the C 1s peak at 284.8 eV). The porosity and specific surface area of the samples were measured by N_2_ adsorption/desorption instrument (Micromeritics ASAP 2020).

### 2.3. Gas sensor Fabrication and Analysis

The gas sensing performance of the samples was measured on an intelligent gas sensing analysis system of CGS-4TPS (Beijing Elite Technology Co., Ltd., Beijing, China), and the gas sensors were fabricated according to our previous method [56]. In a typical sensor fabrication procedure, approximately 10 mg of as-prepared sample was mixed with 1 mL of distilled water by grinding in an agate mortar to obtain a uniform slurry. The slurry was brush-coated on the surface of a ceramic substrate (Al_2_O_3_, size: 13.4 mm × 7 mm × 0.635 mm) with interdigitated Ag-Pd electrodes (width: 0.2 mm, gap distance: 1 mm) and then dried naturally at room temperature to obtain a resistance-type sensor. Before the test, the fabricated sensors were aged at 280 °C for 10 h to improve their stability. By using ambient air as the diluted and reference gas, a static gas distribution method was applied to achieve a desired concentration of target gas in the test chamber (1.8 L). The response of the bare and Ag-modified In_2_O_3_ sensors to the reducing analyte is defined as *R*_a_/*R*_g_, where *R*_a_ and *R*_g_ refer to the resistance of the sensor in air and target gas, respectively. The response and recovery time is measured by recording the time of 90% sensor resistance change after the target gas was injected or released. During the test, the environment temperature was about 25 °C and the relative humidity was 30 ± 8%.

## 3. Results and Discussion

### 3.1. Sample Characterization

**Figure 1:**
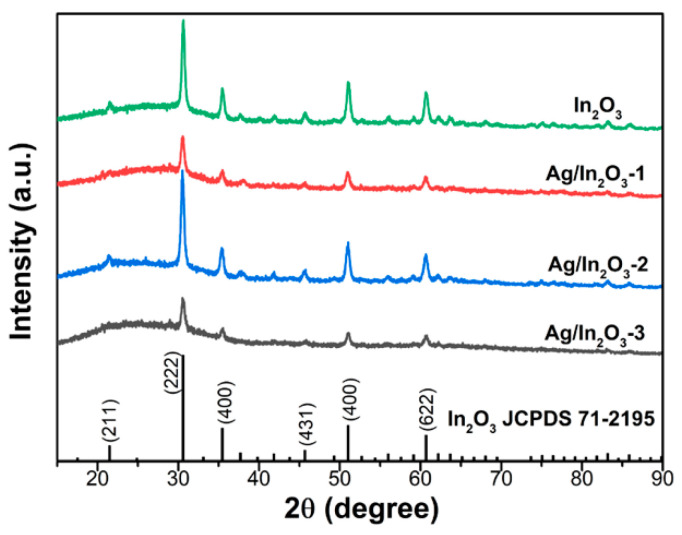
XRD patterns of the prepared bare In_2_O_3_ and Ag-decorated In_2_O_3_ samples.

The phase structure and composition of the prepared samples were confirmed by XRD analysis. As shown in Figure 1, all diffraction peaks match well with the standard data of cubic In_2_O_3_ (JCPDS No. 71-2195, a = b = c = 10.117 Å), demonstrating the formation of the cubic In_2_O_3_ phase in the four samples. The strong and sharp diffraction peaks reveal their good crystallinity. No obvious peaks arising from Ag metal or its related compounds were detected in the Ag/In_2_O_3_ samples. To determine the actual Ag contents in the Ag/In_2_O_3_ samples, ICP-OES measurements were conducted. The results show that the Ag contents in Ag/In_2_O_3_-1, Ag/In_2_O_3_-2, and Ag/In_2_O_3_-3 are 2.43, 2.90, and 3.68 wt%, respectively. Obviously, the actual Ag content of Ag/In_2_O_3_ samples is lower than their designed amount. This phenomenon is similar to that of a previous report [25]. Therefore, the absence of Ag peaks in the XRD patterns should be attributed to the low content of Ag and the sufficient dispersion of Ag nanoparticles in the surface of In_2_O_3_.

**Figure 2:**
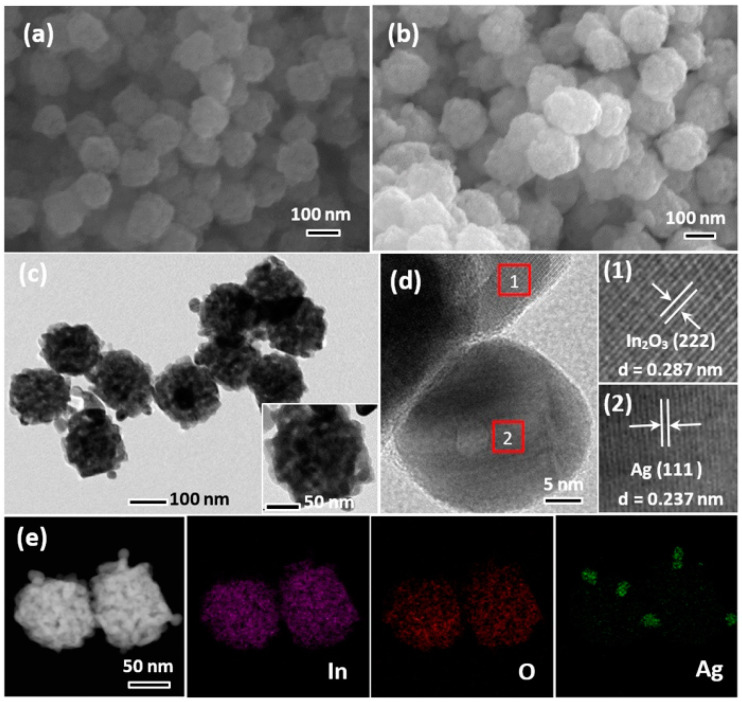
FESEM images of (**a**) bare In_2_O_3_ and (**b**) Ag/In_2_O_3_-2. Typical (**c**) TEM and (**d**) HRTEM images of Ag/In_2_O_3_-2. (**e**) EDS element maps of two adjacent Ag/In_2_O_3_ PNSs observed in Ag/In_2_O_3_-2. The inset in (**c**) is an enlarged TEM image of a single Ag/In_2_O_3_ PNS.

The SEM and TEM were applied to observe the microstructure and morphology of the prepared samples. Figure 2a,b shows the representative SEM images of bare In_2_O_3_ and Ag/In_2_O_3_-2, respectively. Both samples show similar sphere-like structure with the diameter about 120 nm, demonstrating that Ag modification has almost no influence on the basic morphology of primary In_2_O_3_. Figure 2c shows the TEM image of Ag/In_2_O_3_-2, from which the porous structure of the nanospheres is clearly observed. In the high-resolution TEM (HRTEM) image recorded from the edge of a randomly selected nanosphere (Figure 2d), two definite crystalline phases were determined by measuring the lattice fringes. The lattice fringes with the separation distance of 0.287 nm (as displayed in (1) and 0.237 nm (as displayed in (2) correspond to the (222) plane of cubic In_2_O_3_ (JCPDS No. 71-2195) and (111) plane of Ag metal (JCPDS No. 87-0598), respectively. EDS measurement was performed to further confirm the successful decoration of Ag nanoparticles on In_2_O_3_ PNS. In the obtained element maps (Figure 2e), besides the well dispersed In and O elements, agglomerated Ag element was also observed on the nanospheres, strongly demonstrating the decoration of Ag nanoparticles on the In_2_O_3_ PNSs. The average size of the Ag nanoparticles was measured to be about 25–30 nm.

**Figure 3:**
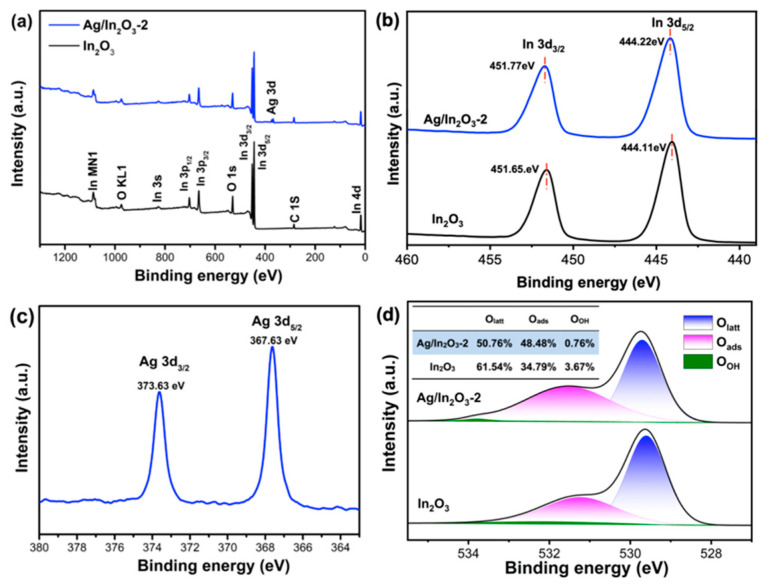
XPS spectra of the In_2_O_3_ and Ag/In_2_O_3_-2: (**a**) full survey spectra, (**b**) high-resolution In 3d spectra, (**c**) high-resolution Ag 3d spectrum of Ag/In_2_O_3_-2, and (**d**) high-resolution O 1s spectra.

The surface chemical state of bare In_2_O_3_ and Ag/In_2_O_3_-2 was investigated by XPS (Figure 3). In their full XPS spectra (Figure 3a), the signals from In, O, and C are clearly observed. The signals of C in the two samples are ascribed to the contaminated carbon. Compared with bare In_2_O_3_, additional signals arising from Ag 3d are presented in Ag/In_2_O_3_-2, further confirming the successful introduction of Ag in In_2_O_3_. Figure 3b shows the high-resolution XPS spectra of In 3d. In the bare In_2_O_3_ sample, the peaks at the binding energy of 451.65 and 444.11 eV belong to In 3d_3/2_ and In 3d_5/2_, respectively. In comparison with bare In_2_O_3_, the In 3d_3/2_ and In 3d_5/2_ peaks of Ag/In_2_O_3_-2 shift slightly to higher binding energy, which are located at 451.77 and 444.22 eV, respectively. The different binding energies of In 3d in the two samples suggest an electronic interaction between Ag and In_2_O_3_ because of their different work functions [57]. In the high-resolution XPS spectrum of Ag 3d of Ag/In_2_O_3_-2 (Figure 3c), the Ag 3d_5/2_ (367.63 eV) and Ag 3d_3/2_ (373.63 eV) peaks with 6 eV splitting demonstrate the existence of Ag^0^ [54,58], being consistent with above TEM analysis. Compared with the standard binding energy of Ag 3d_5/2_ (368.2 eV) and Ag 3d_3/2_ (374.2 eV) in metallic Ag, such two peaks in Ag/In_2_O_3_-2 shift slightly to the lower binding energy side, which can be also ascribed to the interaction of Ag with In_2_O_3_. Figure 3d shows the high-resolution XPS spectra of O1s. After deconvolution, the signals of O1s can be divided into three peaks, including lattice oxygen (O_Latt_) at about 529.60 eV, surface adsorbed oxygen (O_ads_) at about 531.24 eV, and surface OH groups (O_OH_) at about 532.08eV [33,59], and their relative percentages are listed in the inset. The estimated percentage of O_ads_ in Ag/In_2_O_3_-2 is 48.48%, which is higher than that in bare In_2_O_3_ (34.79%). It was generally believed that during the gas sensing process, the surface chemisorbed oxygen species, including O_2_^−^, O^−^, and O^2−^, can play the role of oxidant to react with reducing gas. Therefore, the higher O_ads_ content is usually favorable for enhanced gas sensitivity.

**Figure 4:**
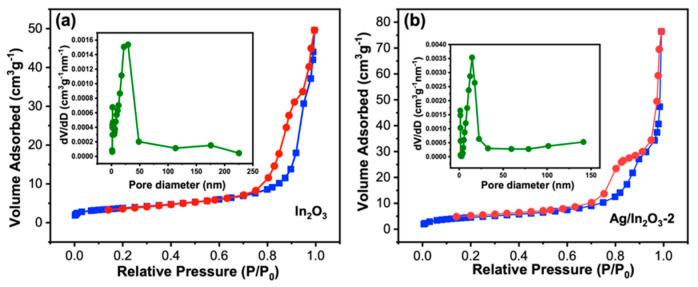
N_2_ adsorption and desorption isotherms and pore-size distribution curves (insets) of (**a**) In_2_O_3_ and (**b**) Ag/In_2_O_3_-2.

The specific surface area and porosity of In_2_O_3_ and Ag/In_2_O_3_-2 were investigated by N_2_ adsorption and desorption method, as shown in Figure 4. Both samples show clear hysteresis loops at P/P_0_ = 0.6–1 and their isotherms increase rapidly at the position near P/P_0_ = 0, revealing the co-existence of micro- and mesoporous structures in In_2_O_3_ and Ag/In_2_O_3_-2. From their pore diameter distribution plots (insets of Figure 4a,b), the dominant diameters of micropore and mesopore are 1.88 and 29.24 nm for bare In_2_O_3_ and 1.17 and 14.63 nm for Ag/In_2_O_3_-2, respectively. Based on the BET method, the calculated specific surface areas of In_2_O_3_ and Ag/In_2_O_3_-2 are 13.20 and 16.39 m^2^g^−1^, respectively.

### 3.2. Gas Sensing Performance

**Figure 5:**
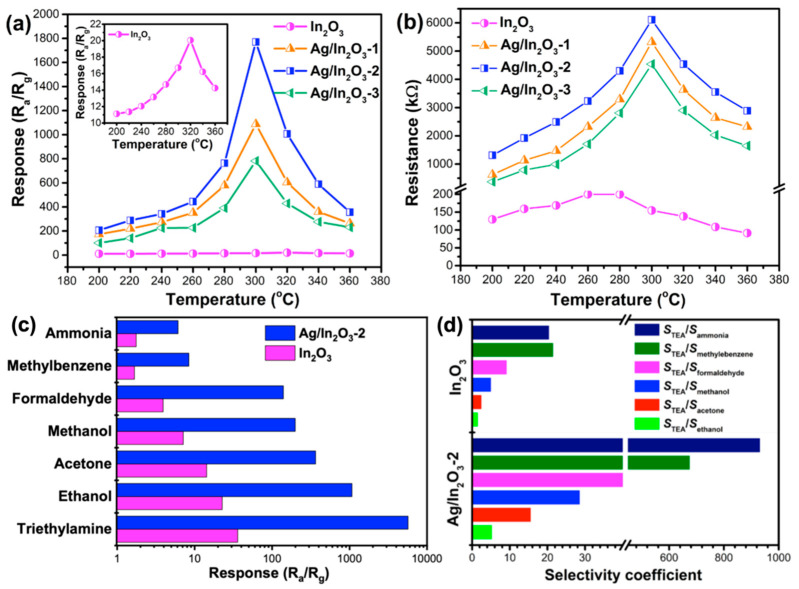
(**a**) Temperature-dependent responses of the sensors to 20 ppm TEA, and (**b**) baseline resistances of the sensors at different operating temperatures. (**c**) Responses of In_2_O_3_ and Ag/In_2_O_3_-2 to different gases (50 ppm) at 300 °C, and (**d**) corresponding selectivity coefficients by using TEA as target gas.

In order to realize an insight into the effects of Ag modification on the TEA sensing performance of In_2_O_3_, gas sensing tests were carried out on the bare and Ag-modified In_2_O_3_ sensors. In view of the fact that the gas sensing characteristics of a MOS sensor greatly rely on its working temperature, the temperature-dependent resistances of the sensors as well as their responses to 20 ppm TEA were first measured. As shown in Figure 5a, with the temperature increasing from 200 to 360 °C, the bare and Ag-modified In_2_O_3_ sensors show similar response variation trends of “increase–maximum–decrease” and reach their maximum responses at 320 and 300 °C, respectively. At different operating temperatures, the Ag/In_2_O_3_ sensors always show higher responses than the In_2_O_3_ sensor, demonstrating the sensitization effect of Ag nanoparticles on In_2_O_3_ PNSs. From Figure 5b, one can see that the sensors’ resistance variation is similar to their response variation. Moreover, in the whole temperature range, the baseline resistances (*R*_a_) of Ag/In_2_O_3_ sensors are always higher than that of bare In_2_O_3_ sensor. Based on the widely accepted oxygen adsorption theory [15,60], the temperature-dependent variation in sensor resistance and response may be associated with the adsorption and desorption behavior of oxygen molecules. When the operating temperature is relatively low (such as 200 °C), less oxygen molecules can adsorb on In_2_O_3_ and Ag/In_2_O_3_ to form chemisorbed oxygen due to their lower activity, which will lead to their lower resistance and response. With the increase in operating temperature, more and more oxygen molecules can acquire enough energy to adsorb on the materials and then transform into chemisorbed oxygen species, resulting in the gradually increased resistance and response of In_2_O_3_ and Ag/In_2_O_3_. The adsorption and desorption of oxygen molecules on the In_2_O_3_ and Ag/In_2_O_3_ PNSs may reach their balance at 280 and 300 °C, respectively. At this moment, the amounts of chemisorbed oxygen species will reach their maximum values, leading to the maximum resistance and response, correspondingly. However, as the temperature exceeds 280 °C for In_2_O_3_ and 300 °C for Ag/In_2_O_3_, the adsorption–desorption balance of oxygen will remove to the desorption side, which will decay the resistance and response due to the decreased amount of chemisorbed oxygen species. In Figure 5b, the higher resistance of Ag/In_2_O_3_ sensors than the bare In_2_O_3_ sensor can be understood from two aspects. Firstly, Ag nanoparticles can facilitate the formation of chemisorbed oxygen on In_2_O_3_ through the spillover effect [61]. Thus, at different operating temperatures, more chemisorbed oxygen was created on Ag/In_2_O_3_ than on bare In_2_O_3_. As is well known, the formation of chemisorbed oxygen on n-type MOS usually accompanies increased sensor resistance because of the decreased carrier (electron) concentration. Thus, more chemisorbed oxygen species can endow Ag/In_2_O_3_ sensors with higher resistance. Secondly, at the interface of Ag and In_2_O_3_, electrons will migrate from In_2_O_3_ to Ag on account of the lower work function of In_2_O_3_ (4.3 eV) than that of Ag (4.6 eV), which will also lead to the higher resistance of Ag/In_2_O_3_ sensors [57]. In addition, from Figure 5a,b, one can clearly observe that the temperature-dependent response variation of all Ag/In_2_O_3_ sensors is strictly in accordance with their resistance variation, and both their response and resistance reach the maximum values at 300 °C, while, in contrast to the Ag/In_2_O_3_ sensors, the bare In_2_O_3_ sensor gives its maximum resistance at 280 °C, which is lower than its maximum response temperature (320 °C). Such a phenomenon is supposed to be relevant to the catalytic effect of Ag nanoparticles. In general, the response of a MOS sensor is mainly controlled by the gas sensing reaction occurring between chemisorbed oxygen and gaseous analyte. As the gas concentration of analyte is fixed, more chemisorbed oxygen species participating in the reaction usually result in higher sensor response. In the Ag/In_2_O_3_ sensors, Ag nanoparticles can catalyze the reaction that occurred between chemisorbed oxygen and TEA, which means that at the temperature before 300 °C, most reactants (chemisorbed oxygen species and TEA molecules) can be activated to complete the gas sensing reaction. In this case, the amount of chemisorbed oxygen species on Ag/In_2_O_3_ becomes the decisive factor of sensor response. Since the amount of chemisorbed oxygen of Ag/In_2_O_3_ sensors reaches the maximum value at 300 °C (Figure 5b), their maximum response to TEA also appears at 300 °C, accordingly. While, for bare In_2_O_3_ sensor, perhaps due to the lack of the catalytic effect of Ag nanoparticles, most of the chemisorbed oxygen species formed at 280 °C could be not active enough to react with TEA until the temperature increases to 320 °C. Therefore, it realizes the highest response at 320 °C but not at 280 °C. From Figure 5b, we can also observe that with the Ag content increases from 2.43 wt% (Ag/In_2_O_3_-1) to 2.90 wt% (Ag/In_2_O_3_-2) and 3.68 wt% (Ag/In_2_O_3_-3), the *R*_a_ values of the sensor first increase and then decease. This phenomenon may be explained in that, when the Ag content is lower than 2.90 wt%, the Ag nanoparticles decorated on In_2_O_3_ have good dispersion. In this case, the existence of more well-dispersed Ag nanoparticles will enhance the spillover effect, resulting in the higher Ra values of Ag/In_2_O_3_-2 than Ag/In_2_O_3_-1. Whereas, with the Ag content further increasing to 3.68 wt% (Ag/In_2_O_3_-3), aggregation between Ag nanoparticles can occur, which will weaken the spillover effect and lead to a lower R_a_ of Ag/In_2_O_3_-3 than Ag/In_2_O_3_-2. Since the Ag/In_2_O_3_-2 sensor shows the highest response among different Ag-modified In_2_O_3_ sensors (Figure 5a), in the following tests, it is chosen as the representative sensor to evaluate the effect of Ag modification on the gas sensing performance of In_2_O_3_ by comparing with bare In_2_O_3_ sensor.

Figure 5c shows the responses of In_2_O_3_ and Ag/In_2_O_3_-2 sensors to 50 ppm various gases at 300 °C. Apparently, the responses of Ag/In_2_O_3_-2 for different gases are obviously higher than that of bare In_2_O_3_, further demonstrating the positive effect of Ag nanoparticles on the gas sensing performance of In_2_O_3_ PNSs. Additionally, both sensors exhibit much higher response to TEA than to other gases, revealing their good selectivity to TEA. To quantitatively evaluate the influence of Ag modification on the TEA selectivity of In_2_O_3_, the TEA selectivity coefficients of In_2_O_3_ and Ag/In_2_O_3_-2 were calculated by using S_TEA_/S_interference gas_, where S_TEA_ and S_interference gas_ refer to the responses of the sensors to TEA and interference gas, respectively. As depicted in Figure 5d, when using different gases as interference gas (ammonia, formaldehyde, methanol, ethanol, acetone, and methylbenzene), the Ag/In_2_O_3_-2 sensor always gives much higher selectivity coefficients than the bare In_2_O_3_ sensor. For example, the values of S_TEA_/S_ethanol_ and S_TEA_/S_ammonia_ of Ag/In_2_O_3_-2 are 5.30 and 932.43, which are about 3.33 and 45.55 times higher than that of In_2_O_3_, respectively. The higher response and better selectivity of Ag/In_2_O_3_-2 to TEA make it more suitable for practical application.

**Figure 6:**
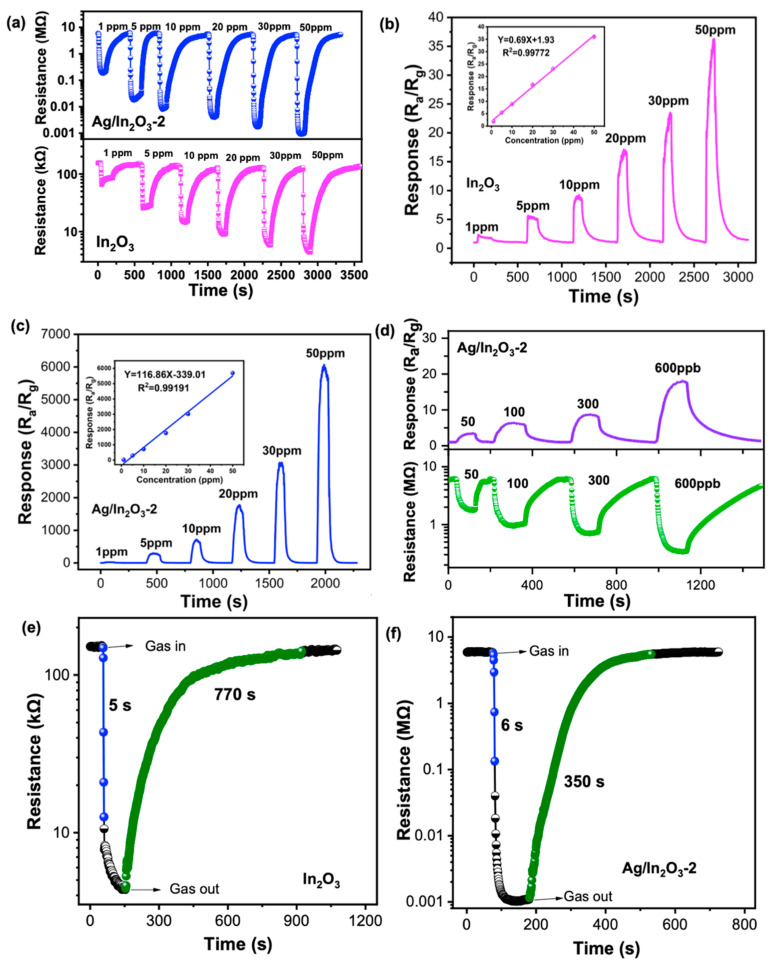
(**a**) Dynamic resistance change in In_2_O_3_ and Ag/In_2_O_3_-2 as exposure to 1–50 ppm TEA at 300 °C. Real-time response–recovery curve of (**b**) In_2_O_3_ and (**c**) Ag/In_2_O_3_-2 for 1–50 ppm TEA at 300 °C (the insets are their corresponding liner relationship between response and gas concentration.). (**d**) Dynamic resistance change and real-time response–recovery curve of Ag/In_2_O_3_-2 as exposure to 50–600 ppb TEA at 300 °C. Transient resistance change in (**e**) In_2_O_3_ and (**f**) Ag/In_2_O_3_-2 as exposure to 50 ppm TEA at 300 °C.

Figure 6a shows the dynamic resistance changes in In_2_O_3_ and Ag/In_2_O_3_-2 as they were exposed to 1–50 ppm of TEA at 300 °C. Both sensors show a rapid decay of resistance once exposure to TEA vapor, exhibiting the characteristic response of n-type MOS. With the increase in TEA concentration, the response amplitudes of the two sensors enlarge gradually (Figure 6b,c), while, for different concentrations of TEA, the Ag/In_2_O_3_-2 sensor gives much higher responses than the bare In_2_O_3_ sensor, demonstrating its superior ability for sensing TEA. For instance, the responses of Ag/In_2_O_3_-2 to 50 ppm TEA are as high as 5697, about 158 times higher than that of bare In_2_O_3_ (36). In the tested TEA concentration range, both the bare In_2_O_3_ and Ag/In_2_O_3_-2 sensors show good response linearity (the insets in Figure 6b,c). Based on the slop of their fitting line, the sensitivity of In_2_O_3_ and Ag/In_2_O_3_-2 to 1–50 ppm TEA is determined as 0.69 and 116.86/ppm, respectively. Apparently, after modification with Ag, the sensitivity of our In_2_O_3_ sensor to TEA is dramatically increased (about 169.36 times). Since the Ag/In_2_O_3_-2 sensor can give a high response (24.3), even to 1 ppm TEA, its response to ppb level TEA was further tested. As shown in Figure.6d, with increasing TEA concertation from 50 to 600 ppb, the sensor shows a gradually increased response. The response to 50 ppb TEA is as high as 3.4, demonstrating its strong sensing ability for TEA at ppb level. The response and recovery speed of bare In_2_O_3_ and Ag/In_2_O_3_-2 were also measured from their transient resistance response–recovery curves to 50 ppm TEA (Figure 6e,f), whose results show that the response/recovery time (τ_res._/τ_rec._) of In_2_O_3_ and Ag/In_2_O_3_-2 are 5/770s and 6/350s, respectively. Perhaps because of their relatively higher operating temperature, both sensors can give fast response to TEA, while, compared with bare In_2_O_3_ sensor, the Ag/In_2_O_3_-2 sensor shows much shorter recovery time. Theoretically, the recovery time of a MOS sensor to garget gas is mainly controlled by two factors, including the desorption speed of the product of gas sensing reaction and the regeneration speed of chemisorbed oxygen on the surface of MOS. In our case, the faster recovery speed of Ag/In_2_O_3_-2 may mean that Ag nanoparticle can not only accelerate the regeneration of chemisorbed oxygen through its strong spillover effect, but also can catalyze the gas sensing reaction to produce some products that can easily desorb from In_2_O_3_ PNS. To further evaluate the quality of Ag/In_2_O_3_-2, its TEA sensing performances were compared with that of the previously reported sensors. As displayed in Table 1, the present Ag/In_2_O_3_-2 sensor shows much higher response and better selectivity to TEA than most of the reported sensors, demonstrating its superiority in TEA detection.

**Figure 7:**
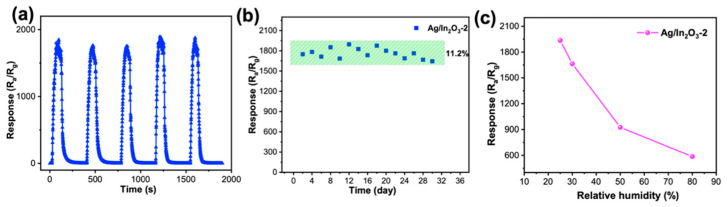
(**a**) Repeatability and (**b**) long-term stability test of Ag/In_2_O_3_-2 for 20 ppm TEA; (**c**) responses of Ag/In_2_O_3_-2 to 20 ppm TEA under different relative humidity (RH).

The repeatability, long-term stability, and humidity resistance of the Ag/In_2_O_3_-2 sensor were also tested to further evaluate its quality. As shown in Figure 7a,b, in five continues response–recovery cycles test results, the sensor can give almost the same response amplitudes to 20 ppm TEA; moreover, within a period of 30 days, there is only a slight decay in its response, revealing its good repeatability and stability. Figure 7c presents the response values of Ag/In_2_O_3_-2 sensor toward 20 ppm TEA under different relative humidity (RH). With increasing RH from 25 to 80%, the response of the sensor decreases seriously. Under humidity conditions, water molecules can absorb on the surface of Ag/In_2_O_3_ PNSs and then compete with TEA molecules to react with chemisorbed oxygen [25], resulting in a decreased response of TEA under high humidity. According to the research results of Lee’s group, doping our In_2_O_3_ PNSs with Pr before modification with Ag nanoparticles may be a possible strategy to improve the humidity resistance of the present Ag/In_2_O_3_ sensor [65], which will be tried in our future work.

### 3.3. Gas Sensing Mechanism

**Figure 8:**
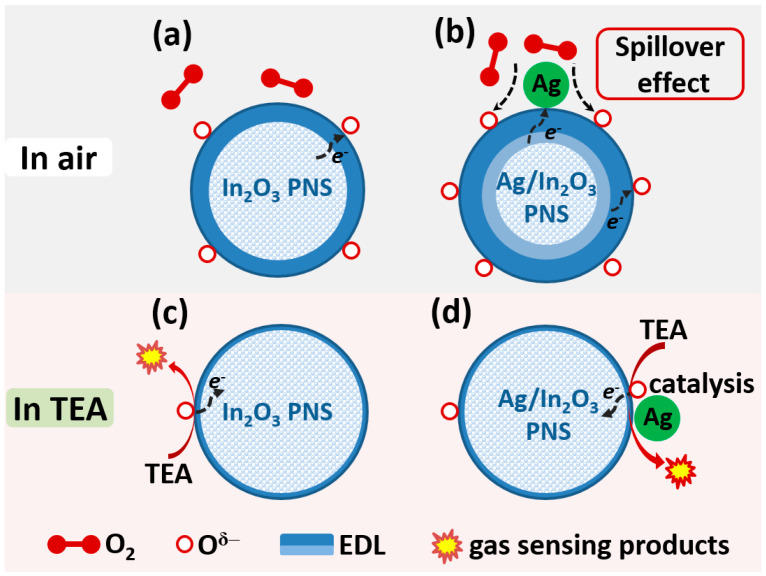
Schematic illustration for the enhanced TEA sensitivity of In_2_O_3_ PNS by modifying with Ag nanoparticles.

In_2_O_3_ is a typical n-type MOS, and its gas sensing phenomenon can be illustrated by widely accepted oxygen adsorption theory [66,67]. As schematically illustrated in Figure 8, when our In_2_O_3_ PNS sensor is exposed in air atmosphere, oxygen molecules can adsorb on the surface of In_2_O_3_ PNS and then capture electrons from its conduction band to form chemisorbed oxygen species (Equations (1)–(4)). As a result, a thick electron depletion layer (EDL) with higher resistance will form on the surface of In_2_O_3_ PNS, leading to its higher sensor resistance in air (*R*_a_) (Figure 8a). Meanwhile, as the sensor is exposed to reducing gases, such as TEA, a redox reaction (gas sensing reaction) between TEA and chemisorbed oxygen species will occur, after which the electrons captured by chemisorbed oxygen will be released back to In_2_O_3_, resulting in a thinner EDL and a decreased sensor resistance (*R*_g_), accordingly (Figure 8c). The varied resistance of the In_2_O_3_ PNS sensor in air and target gas endows it with the gas sensing ability.

O_2(*gas*)_→O_2(*ads*)_ (1)

O_2(*ads*)_ + *e*^−^→O_2_^−^_(*ads*)_ (2)

O_2_^−^_(*ads*)_ + *e*^−^→2O^−^_(*ads*)_ (3)

O^−^_(*ads*)_ + *e*^−^→O^2−^_(*ads*)_ (4)

**Figure 9:**
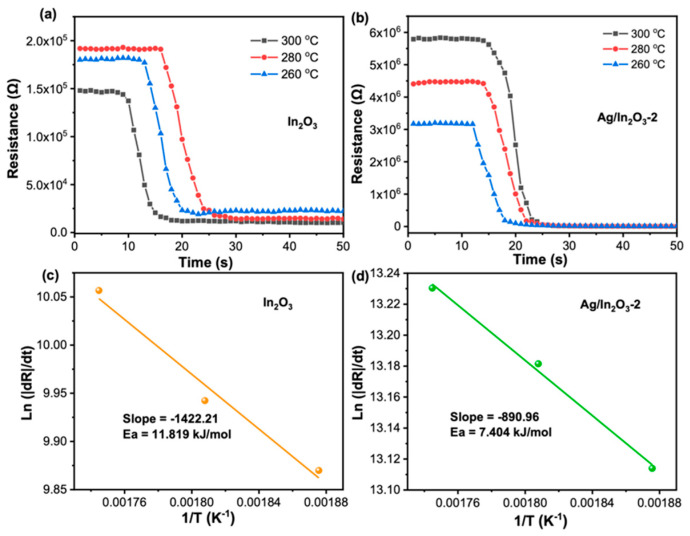
The transient resistance change of (**a**) In_2_O_3_ and (**b**) Ag/In_2_O_3_-2 as exposure to 20 ppm TEA at different temperatures, and derived Arrhenius-type plots of (**c**) In_2_O_3_ and (**d**) Ag/In_2_O_3_-2.

In our experiment, the Ag/In_2_O_3_-2 sensor, like the bare In_2_O_3_ sensor, shows the characteristic response of an n-type MOS, but its gas sensing performance to TEA was remarkably boosted, especially in terms of lower operating temperature, higher sensitivity, and better selectivity. These improvements in gas sensing performance can be mainly attributed to the sensitization effects of Ag nanoparticles on In_2_O_3_ PNSs. Firstly, Ag nanoparticle can promote the dissociation of oxygen molecules to form chemisorbed oxygen through its strong “spillover effect” [53] (Figure 8c). That is to say, in air atmospheres, more chemisorbed oxygen species (O^2−^) will be created on Ag-decorated In_2_O_3_ PNSs than on the bare In_2_O_3_ counterpart (the XPS spectra of O 1s in Figure 3d indicate that Ag/In_2_O_3_-2 owns higher O_ads_ content than bare In_2_O_3_, supporting this speculation). On one hand, the increase in chemisorbed oxygen can endow the Ag/In_2_O_3_-2 sensor with higher *R*_a_ value (Figure 5b), which is favorable for higher response because the response of the Ag/In_2_O_3_ sensor to TEA is defined as *R*_a_/*R*_g_. On the other hand, it will promote the surface sensing reaction due to the increased concentration of O^2−^ reactant, which also contributes to the enhancement in TEA response. Additionally, the faster recovery speed of Ag/In_2_O_3_-2 (Figure 6f) may also be relevant to the “spillover effect” of Ag nanoparticles, which can accelerate the regeneration of chemisorbed oxygen after switching the sensor from TEA to air ambient. Secondly, Ag has good catalytic property. When decorating Ag nanoparticles on In_2_O_3_ PNS, they can catalytic the gas sensing reaction that occurs between TEA and chemisorbed oxygen species (Figure 8d), leading to a lower reaction barrier and a decreased operating temperature, accordingly. As in our discussion on Figure 4a,b, due to the catalytic effect of Ag nanoparticles, the Ag/In_2_O_3_ sensors show the maximum resistance and response at the same temperature (300 °C); in contrast, the bare In_2_O_3_ sensor shows higher temperature of maximum response (320 °C) than that of its maximum resistance (280 °C). Such a difference between bare In_2_O_3_ and Ag/In_2_O_3_ sensors supports, to some extent, the existence of catalytic effect of Ag nanoparticles in the Ag/In_2_O_3_ sensors. To prove above speculation, the apparent activation energies (*E*_a_) of bare In_2_O_3_ and Ag/In_2_O_3_-2 were estimated by their Arrhenius-type plots (Figure 9c,d) that derived from Figure 9a,b [17,61]. The results show that the *E*_a_ values of bare In_2_O_3_ and Ag/In_2_O_3_-2 are 11.819 and 7.404 kJ/mol, respectively. The lower *E*_a_ of Ag/In_2_O_3_-2 than that of bare In_2_O_3_ convinces the existence of catalytic effect of Ag, which not only endows the Ag/In_2_O_3_-2 sensor with lower OWT, but also endows it with higher response for sensing TEA. Thirdly, when decorating Ag nanoparticles on the surface of In_2_O_3_ PNSs, the Schottky junction between Ag and In_2_O_3_ will be created. Since the work function of In_2_O_3_ (4.3 eV) is smaller than that of Ag (4.6 eV) [57], at the interface of Ag nanoparticles and In_2_O_3_ PNSs, electrons will flow from In_2_O_3_ to Ag to equilibrate their Fermi level (Figure 8b). Consequently, on the In_2_O_3_ side, besides the EDL caused by chemisorbed oxygen, an additional EDL will be also formed, leading to a thicker EDL on Ag/In_2_O_3_ and a higher response than bare In_2_O_3_ accordingly.

In addition, both our In_2_O_3_ and Ag/In_2_O_3_ sensors show good selectivity to TEA. Generally, the gas selectivity of a MOS sensor can be influenced by many factors, such as the adsorption property of target gas on the surface of MOS, the reactivity of reactants (chemisorbed oxygen species and adsorbed target gas), the surface catalytic property of MOS, and so on. In our case, the relatively lower bond energy of C-N should be one of the possible reasons for the good TEA selectivity of the In_2_O_3_ and Ag/In_2_O_3_ sensors. The bond energy of C-N in TEA is 307 kJ/mol, which is lower than that of C=C (toluene, 610.3 kJ/mol), N-H (ammonia, 386 kJ/mol), H-O (ethanol and methanol, 458 kJ/mol), and C=O (formaldehyde and acetone, 798.9 kJ/mol) [61]. The lowest bond energy of C-N endows TEA molecules with high reaction activity during the gas sensing process, leading to good TEA selectivity.

## 4. Conclusions

In summary, well-dispersed Ag nanoparticles with a size of about 25–30 nm were decorated on In_2_O_3_ PNSs (~100 nm in diameter) via a room-temperature chemical reduction method. After modification with Ag nanoparticles, the In_2_O_3_ PNSs sensor showed remarkable improvements in TEA sensing performance, especially of lower OWT (decreased from 320 to 300°C), higher sensitivity (increased from 0.69/ppm to 116.86/ppm for 1–50 ppm TEA), and better selectivity (S_TEA_/S_ethanol_ increased from 1.59 to 5.30). These improved gas sensing performances of as-prepared Ag/In_2_O_3_ were contributed to the positive effects of Ag, the spillover effect, and electron sensitization of the Ag–In_2_O_3_ Schottky junction, as well as the catalysis effect of Ag on the surface gas sensing reaction between TEA and chemisorbed oxygen. Our research strongly demonstrates that modification of In_2_O_3_ with Ag is a promising strategy to develop an advanced TEA sensor.

## Figures and Tables

**Table 1:** Compared TEA sensing performances of different materials.

Materials	Con. (ppm)	Response (R_a_/R_g_)	Temp. (°C)	Tres/Trec (s)	S_TEA_/S_ethanol_	Ref.
Ag/In_2_O_3_ porous nanosphere	50	5697.15	300	6/350	5.30	This work
Single crystalline In_2_O_3_ nanoplates	50	7.8	320	10/19	5.20	[62]
SnO_2_: Ho^3+^ nanoparticles	50	12	175	2/120	1.88	[63]
Zn-doped In_2_O_3_ nanospheres	50	36	280	9/7	3.00	[18]
Yolk-shell SnO2/Au/Fe_2_O_3_ nanoboxes	100	126.84	240	7/10	1.41	[20]
Pd NPs-In_2_O_3_	50	47.56	220	4/17	1.85	[52]
Au@SnO_2_/α-Fe_2_O_3_ nanoneedles	100	39	300	4/203	2.57	[64]
